# Presumed Acute Pancreatitis During Chickenpox Infection in an Otherwise Healthy Male

**DOI:** 10.4103/1319-3767.39631

**Published:** 2008-04

**Authors:** Roberto Manfredi

**Affiliations:** University of Bologna, C/O Infectious Diseases, S. Orsola Hospital, Via Massarenti 11, 40138 Bologna, Italy. E-mail: roberto.manfredi@unibo.it

Sir,

Kumar ***et al.***[1] reported a rare case of fatal acute pancreatitis attributed to chickenpox infection in an otherwise healthy young man. This intriguing case report poses a series of challenging questions, and eventual authors’ responses and clarifications are expected to add significantly to our limited knowledge in this field.

End-organ complications infrequently occur during primary, acute Varicella Zoster virus infection and may manifest via somewhat different pathogenetic pathways, including a direct viral damage or an indirect (usually immune-mediated) mechanism or a superinfection (usually bacterial in origin).

Among the few presumed predisposing conditions, the authors excluded the childhood[2] and the frankly immunocompromised patients, like those with AIDS, and those who had undergone bone marrow or solid-organ transplantation. On the other hand, it is well recognized that even severe chickenpox complications may occasionally occur in otherwise healthy people and especially subjects with unfavorable supporting conditions such as extreme old age, pregnancy, low-dose (including topical) corticosteroid therapy, and minor immunodeficient conditions.

Isolated cases of chickenpox-related acute pancreatitis have been anecdotally observed since nearly 50 years, especially in children, as per the global epidemiological records of chickenpox support,[2] but only in rare occurrences, the exact pathogenesis has been elucidated. This is because histopathological specimens are usually needed to distinguish acute viral damage due to an immune-mediated reaction from a superinfection or a multiorgan involvement. In most of these cases, unfortunately only necropsy studies clarified the pathogenesis and explained the single organ damage mechanisms.

An increased awareness of the pathogenesis of pancreatic damage may also have a significant impact in evaluating the role and timeliness of specific antiviral therapy, which is usually expected to add something only when started in the first 24–48 h of disease commencement[3,4] and seems to reduce the incidence and/or the severity of organ complications, especially when a direct, viral-mediated damage is of concern.

With regard to the described patient, it could be very interesting to have some information on the predisposing factors: was the increased body mass index (obesity) associated with hyperlipidemia? and did it serve as a potential risk factor for pancreatitis? Were the prior serum amylaselipase levels measured? Did it lead to any significant finding? And were there any concomitant medications?

Significantly, hepatic-biliary diseases were not evident at the time of occurrence of pancreatic complications, as assessed by biochemistry assays and abdominal imaging studies, although the more elevated serum amylase and lipase levels (907 and 562 U/L, respectively), did not reproduce those expected during hyperacute necrotizing hemorrhagic pancreatitis. Once again, histopathological and immunohistochemical data (eventually from the necropsy examination) are of interest for clarifying the etiopathogensis of pancreatic damage. They can also be used to correlate pancreatic damage with generalized multiorgan failure (seen more frequently in childhood and when an underlying immunodeficiency is of concern), which seems to have rapidly led to the death of the described patient. However, a direct viral damage cannot be excluded, since the authors suspected myocarditis and respiratory distress syndrome along with a probable liver involvement with an apparently inexplicable obstructive jaundice.[1] Also, for these complications, only histopathological studies and direct viral search by immunocytochemical assays, might clarify the pathogenesis and the exact role of Varicella Zoster virus infection in this series of not necessarily related complications. Among others, varicella vaccination has also been sporadically associated with the occurrence of acute pancreatitis.[5]

From a therapeutic point of view, some more clarity regarding antiviral therapy (time of start, dosage, and mode of administration) may be very interesting as well as useful, especially in the event that a direct (viral) pancreatic damage was suspected and possibly confirmed, to assess the role of treatment with acyclovir and its derivatives in such an hyperacute, life-threatening condition.

Finally, until the pathogenetic association has not been proven and the role of eventual co-factors has been carefully ruled out, the authors should refrain discussing “acute pancreatitis due to chickenpox.” This last condition has been reported more frequently when compared with the only two adult otherwise healthy cases recorded by the authors, [6–10] although the clinical background was very different in each of the described cases.

In conclusion, the interesting case report presented by Kumar and colleagues may add even more to the knowledge of pathogenesis of viral tropism, the development of life-threatening multiorgan failure and the eventual role of specific antiviral therapy, should more details be added and discussed on the background of an updated literature review.
